# Variations in Static Force Control and Motor Unit Behavior with Error Amplification Feedback in the Elderly

**DOI:** 10.3389/fnhum.2017.00538

**Published:** 2017-11-08

**Authors:** Yi-Ching Chen, Linda L. Lin, Yen-Ting Lin, Chia-Ling Hu, Ing-Shiou Hwang

**Affiliations:** ^1^Department of Physical Therapy, College of Medical Science and Technology, Chung Shan Medical University, Taichung City, Taiwan; ^2^Physical Therapy Room, Chung Shan Medical University Hospital, Taichung City, Taiwan; ^3^Institute of Physical Education, Health and Leisure Studies, National Cheng Kung University, Tainan City, Taiwan; ^4^Physical Education Office, Asian University, Taichung City, Taiwan; ^5^Department of Physical Therapy, College of Medicine, National Cheng Kung University, Tainan City, Taiwan; ^6^Institute of Allied Health Sciences, College of Medicine, National Cheng Kung University, Tainan City, Taiwan

**Keywords:** motor control, force fluctuations, visuomotor processing, aging, electromyography

## Abstract

Error amplification (EA) feedback is a promising approach to advance visuomotor skill. As error detection and visuomotor processing at short time scales decline with age, this study examined whether older adults could benefit from EA feedback that included higher-frequency information to guide a force-tracking task. Fourteen young and 14 older adults performed low-level static isometric force-tracking with visual guidance of typical visual feedback and EA feedback containing augmented high-frequency errors. Stabilogram diffusion analysis was used to characterize force fluctuation dynamics. Also, the discharge behaviors of motor units and pooled motor unit coherence were assessed following the decomposition of multi-channel surface electromyography (EMG). EA produced different behavioral and neurophysiological impacts on young and older adults. Older adults exhibited inferior task accuracy with EA feedback than with typical visual feedback, but not young adults. Although stabilogram diffusion analysis revealed that EA led to a significant decrease in critical time points for both groups, EA potentiated the critical point of force fluctuations $<\Delta F_{c}^{\text{2}}>$, short-term effective diffusion coefficients (Ds), and short-term exponent scaling only for the older adults. Moreover, in older adults, EA added to the size of discharge variability of motor units and discharge regularity of cumulative discharge rate, but suppressed the pooled motor unit coherence in the 13–35 Hz band. Virtual EA alters the strategic balance between open-loop and closed-loop controls for force-tracking. Contrary to expectations, the prevailing use of closed-loop control with EA that contained high-frequency error information enhanced the motor unit discharge variability and undermined the force steadiness in the older group, concerning declines in physiological complexity in the neurobehavioral system and the common drive to the motoneuronal pool against force destabilization.

## Introduction

Impairment of force steadiness control is linked to unsafe visuomotor tasks in older adults (Lodha et al., 2016). For degenerative changes in the neuromuscular system, force output of the elderly manifests with greater size (Vieluf et al., 2015) and regularity (Vaillancourt and Newell, 2003; Sosnoff and Newell, 2008) in force fluctuations. An age-related increase in force fluctuations indicate impairment of precision force control for force production (Temprado et al., 2017), partly because prolonged delay in a feedback loop interferes with timely responses to tracking deviation (Kennedy and Christou, 2011; Tracy et al., 2015). The force regularity increment with aging supports a loss of physiological complexity in the neurobehavioral system (Vaillancourt and Newell, 2003; Rey-Robert et al., 2011), pertaining to ineffectiveness of using visual information for force gradation (Ofori et al., 2010). As force fluctuations reflect an additive accuracy control mechanism to remedy trajectory deviations (Slifkin et al., 2000), dimensional changes in force fluctuations with advanced age links to a decline in error detection with feedback and/or feedforward processes (James and Kooy, 2011) depending on the environmental contexts (Morrison and Newell, 2015). From the neurophysiological aspect, age-related increase in force fluctuations is primarily attributable to increase in motor unit discharge variability (Laidlaw et al., 1999; Tracy and Enoka, 2002; Tracy et al., 2005), underlying altered properties of spinal motor neurons and greater variability of descending drive to motor neurons (Hunter et al., 2016).

Several recent studies have claimed that virtually displaying worse outcomes, or error amplification (EA) feedback, promises to better optimize a visuomotor task than does actual feedback. Visual EA is thought to inflate response conflicts and facilitate attentional focus on the motor task via error-monitoring networks (Calhoun et al., 2002). The EA feedback has been shown to improve point-to-point visuomotor tasks for healthy elderly (Bouchard et al., 2015) and neurological patients (Abdollahi et al., 2014), as well as a continuous static force-tracking task for young adults (Hwang et al., 2017). For young adults during static isometric contraction, visual EA could better stabilize force output with the enhanced complexity of force fluctuations, corollary to a larger coefficient of variation for inter-spike intervals and a greater motor unit coherence at 13–35 Hz (Hwang et al., 2017). In addition, EA produces a strategic feedback-feedforward mode shift during sinusoidal force tracking (Chen et al., 2017). Visual EA suppressed the phasic discharge of motor units with the target signal, in favor of the use of a feedback process to steer force control. However, we must not be overoptimistic toward EA, especially when the older adults perform a continuous visuomotor task. The reason is apparent that older adults might not be able to exploit rich spatial information in visual feedback to optimize force control (Kennedy and Christou, 2011; Jordan et al., 2013; Park et al., 2017). In fact, age differences in force variability appeared to be minimal in the absence of visual feedback (Tracy et al., 2007). It cannot be denied that the EA could lead to information overload for the elderly considering impaired visuomotor processing. Besides, older adults are less capable of inhibiting functionally-irrelevant information (noise) that is concurrently exaggerated by amplification process (James and Kooy, 2011).

As the effect of visual EA on force control for the older adults could not be emanated directly from the theoretical realm, this study aimed to examine whether EA feedback could improve the force control of a low-level static force task for healthy elderly participants in both behavioral and neural aspects. In particular, we specified paradigm shifts in the feedback and feedforward processes via force fluctuation dynamics and characterized variations in motor unit discharge with multi-electrode surface electromyography (EMG) technology and decomposition procedures. When visual feedback consisted of rapid changes in error information (such as involuntary tremulous movements), unlike the young adults, it was hypothesized that: (1) the elderly might not benefit from EA to stabilize force on account of their relative reliance on a degenerated feedback system; (2) the elderly might exhibit enhancement of discharge variability and reduction in discharge complexity of motor units with high-frequency EA; and (3) the elderly using high-frequency EA might exhibit a decline in motor unit coherence at 13–35 Hz critical to maintain steady-state force control with precision. Our findings are of values for the use of video tracking systems to train visuomotor skills for older adults.

## Materials and Methods

### Participants

Fourteen young adults (7 males and 7 females; mean age: 24.9 ± 0.7 years) and 14 older adults (6 males and 8 females; mean age: 68.2 ± 1.0 years) volunteered to participate in this study. All participants were right-handed with normal or corrected vision, and none had symptoms of neurological or neuromuscular diseases. This study was approved by Institutional Review Board (IRB) at the National Cheng Kung University (NCKU) Hospital, Taiwan. All subjects gave and signed written informed consent in accordance with the Declaration of Helsinki.

### Experimental Procedures

The participants performed force-tracking tasks under error feedback conditions with a normal amplification factor (the control condition) and a high amplification factor (the error enhancement condition). Each condition contained three experimental trials interleaved with 3-min pauses, and all experimental trials were executed in a randomized order. The force-tracking tasks required the participants to couple a static low-level force (20% of maximal voluntary contraction (MVC)) with isometric index abduction on a 27-inch monitor (spatial resolution: 1920 × 1080 pixels). Prior to the experiment, the MVC of the first dorsal interosseus was measured after three 5-s contraction trials separated by 3-min pauses. The highest force value of each trial was averaged to obtain the MVC.

**Figure 1:**
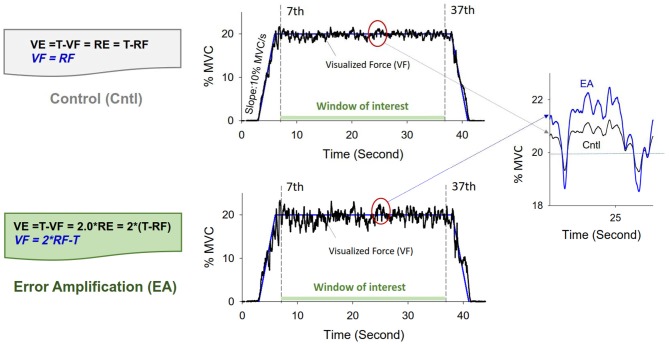
Illustration of modulation of error augmentation feedback. With mathematical transformation, the VF outputs in the error amplification (EA) condition virtually double the size of the real execution error displayed in the control condition. In the control condition, the participant was provided with real error (VE = RE), as the VF output was identical to RF output. In the EA condition, the force output displayed on the monitor (VF) was transformed with VF = 2*RF − T. The size of the perceived tracking error was augmented by two times in the EA condition (VE = T − VF = T − (2*RF − T) = 2*(T − RF) = 2*RE). For a given experimental trial, the rightest plot displays VF at about 25th second in the control (black line) and EA (blue line) conditions. VF, visualized force; VE, visualized error; RF, real force; RE, real error; T, target signal.

After a rest period of 20 min of the MVC test, all the participants were given three practice trials in each feedback condition. They were instructed to produce an isometric force by pushing their index finger against a force transducer (Model: MB-100, Interface Inc., Atlanta, GA, USA) connected to a custom-made amplifier (gain = 10, cut-off frequency = 20 Hz). The spatial resolutions (25 pixels per 1% MVC) of the display target signal and force output were identical for the two experimental conditions. The representation of visual resolution was designed to minimize the effects of visual angle and the group-dependent strength difference on visual information (Kennedy and Christou, 2011). The participants were instructed to reach the target force within 2 s after a latent period of 3 s and then to maintain the force at 20% of MVC for 34 s (Figure 1). It took 44 s to complete an experimental trial. The time window of interest was the 7th to 37th seconds of the experimental trial, during which force output was presumed to be relatively stable. The participants were guided with perceived errors in the control and EA conditions (Chen et al., 2017; Hwang et al., 2017). In the EA condition, the visualized force (VF) output was mathematically transformed so that the size of the visualized error (VE) was virtually augmented by 100% as compared to real error (RE) (VE = 2*RE) (Figure 1). The use of EA factor of two was empirically determined in the laboratory, because an excessive EA factor could cause visual perceptual conflicts and visual information overload. In the control condition, the participants were provided with RE (VE = RE). However, the participants in this study could perceive more execution errors of higher frequency components than could participants in our previous work, which did not include force components above 6 Hz to calculate the VEs. The inclusion of higher frequency components (6–20 Hz) in this study was expected to increase the information load in the EA condition. The real force (RF) output was sampled at 1 kHz with a custom program on a LabVIEW platform (National Instruments Inc., Austin, TX, USA).

### Electromyographic Recordings and Decomposition

Muscle activity of the first dorsal interosseus was recorded with a multi-electrode surface EMG system (Bagnoli sEMG system, Delsys Inc., Natick, MA, USA). The analog EMG signals from each pin-sensor were amplified (gain = 1000) and filtered with a bandwidth of 20–450 Hz (De Luca et al., 2015a). A high sampling rate of 20 KHz was used to avoid phase skew across EMG channels (De Luca et al., 2006). The binary spike events of motor unit discharge that coded the activations of all motor units with values of 0 or 1 were characterized after EMG decomposition using EMG works v.4.1 (Delsys Inc., Natick, MA, USA), according to a previous proof-of-principle using an artificial intelligence framework (De Luca et al., 2006; Nawab et al., 2010). The most recent studies have repeatedly shown that the computation algorithm can produce convincing decomposition results that discriminate overlapping action potentials for static isometric contraction (Nawab et al., 2004; De Luca et al., 2015b) via independent verification methods (Hu et al., 2013). The validity of the EMG decomposition for each motor unit was assessed with the Decomposition-Synthesis-Decomposition-Compare test (De Luca et al., 1982, 2006), and motor units of low decomposition accuracy (<90%) were discarded. Previous studies have reported that the averaged decomposition accuracy using the same algorithm ranges from 92.5% to 97.6% (De Luca et al., 1982, 2006).

### Data Analysis

**Figure 2:**
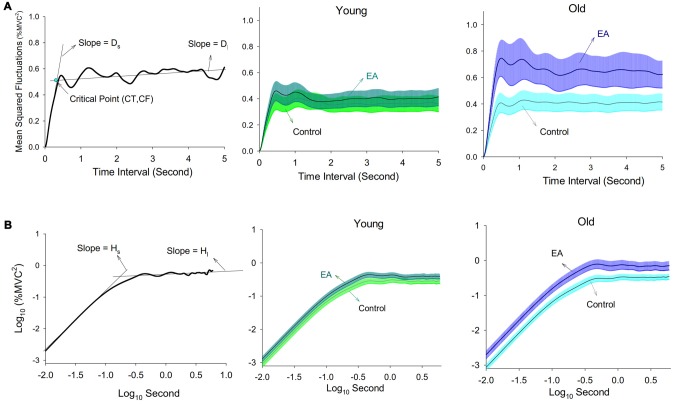
Diffusion plot. **(A)** The left plot is a typical linear-linear diffusion plot. The computed critical point (∆*t_c_*, $<\Delta F_{c}^{\text{2}}>$), short-term effective diffusion coefficient (D_s_), and long-term effective diffusion coefficient (D_l_) are labeled. The critical point indexes the turning point of variation in open-loop and closed-loop control of the stochastic dynamics for force fluctuations. The middle and right plots are pooled linear-linear diffusion plots of the young and old adults. **(B)** The left plot is a typical log-log diffusion plot. The computed short-term scaling exponent (H_s_) and long-term scaling exponent (H_l_) are labeled. The middle and right plots are pooled log-log diffusion plots of the young and old adults.

The force signal was conditioned with a low-pass filter (cut-off frequency: 6 Hz). The resulting force was susceptible to most visuo-motor processes (Slifkin et al., 2000; Vaillancourt et al., 2002) and down-sampled at 100 Hz (Figure 1). In terms of % MVC, the total error was the root mean square (RMS) value of mismatches between the target signal and RF output. The constant error was the difference between the averaged force output and the target signal. The size of force fluctuations was defined as the RMS value of the RF output after removal of a linear trend. Force fluctuations were assumed to be wide-sense stationary (time-invariant mean and auto-covariance; Collins and De Luca, 1993), so the stabilogram diffusion analysis was used to characterize the force fluctuation dynamics by the power-law relationship between the mean-squared value of the force fluctuations (<∆*F*^2^>) and the time interval (∆*t*) in which these values occurred. The stabilogram diffusion analysis can detect parametric shifts of open-loop and closed-loop controls for a posture system due to aging (Addamo et al., 2010; Toosizadeh et al., 2015) and visual information changes (Collins and De Luca, 1995). The Stabilogram diffusion analysis was calculated by using the following equation: $\langle \Delta F^{2}\rangle \;=\;\langle {\left[x\left(t+\Delta t\right)-x\left(t\right)\right]}^{2}\rangle \text{ ,}$ where <●> indicates the mean of the time series. The computation of ∆*F*^2^ was repeated with increasing ∆*t* values ranging from 0 s to 5 s. The diffusion plot was the mean square force fluctuations <∆*F*^2^> against the time intervals ∆*t* (Figure 2A). The regression slopes for the short-term and long-term regions of the diffusion plot were two effective diffusion coefficients (D_s_ and D_l_) that described the stochastic activity of the open-loop and closed-loop force control mechanisms, respectively. The critical point of time (∆*t_c_*) was the intersection of the two regression lines of the diffusion plot, and variations in the critical point of force fluctuations ($<\Delta F_{c}^{\text{2}}>$) reflected a paradigm shift between open-loop and closed-loop behaviors (Collins and De Luca, 1993; Toosizadeh et al., 2015). The short-term and long-term scaling exponents (H_s_ and H_l_) were linear fits of the log–log plot of the stabilogram diffusion analysis (Figure 2B). A scaling exponent greater than 0.5 indicates that the system is governed by the open-loop process and that the data series of the past and future are positively correlated (Collins and De Luca, 1993, 1995). Conversely, a scaling exponent smaller than 0.5 indicates that the data series of the past and future are negatively correlated, as regulated by the closed-loop process.

**Figure 3:**
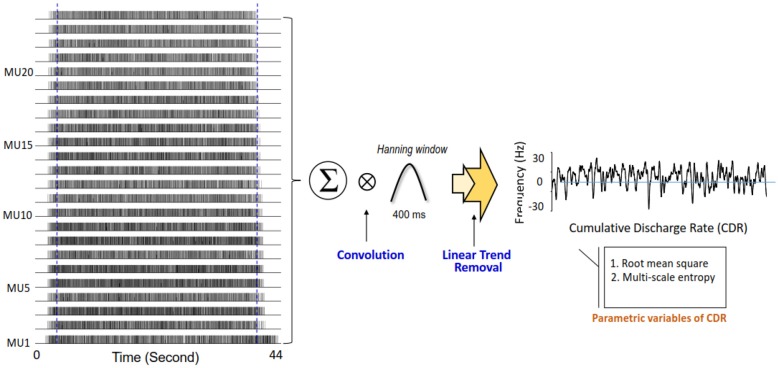
Feature extraction of cumulative discharge rate. Cumulative discharge rate is summation of all motor unit spike trains following smoothing process and removal of linear trend. The size and complexity of cumulative discharge rate are represented with root mean square (RMS) and multi-scale entropy (MSE).

Decomposition processing on the overall EMG data of 44 s was used to identify binary spike trains that coded the timing of motor unit discharges (Figure 3). The mean inter-spike interval of a motor unit was the averaged time intervals of the motor unit spike train in the time window of interest, and the grand average of mean ISI (ISI_GAV_) was the average mean inter-spike interval for all motor units. The variability of the inter-spike interval for an individual motor unit was characterized by the coefficient of variation (ISI CV). The grand average of ISI CV (ISI CV_GAV_) was the population mean of the ISI CV of all motor units. As force behaviors are tuned to pooled motor unit discharge, we estimated cumulative discharge rate by convolution of the cumulative spike trains of all identifiable motor units with a Hanning window of 400 ms in duration (Figure 3). The resulting low-frequency cumulative discharge rate reflects an effective neural drive to a muscle, and force fluctuation dynamics can be largely featured with cumulative discharge rate during static isometric contraction (Negro and Farina, 2012; Farina et al., 2016). The size and complexity of the cumulative discharge rate variability was determined with RMS and multi-scale entropy (MSE) following down-sampling process at 100 Hz and removal of its linear trend. The MSE accounts for complexity of signals over different time scales, proposed by Costa et al. (2005). Based on the sample entropy, this method quantifies the regularity of a time series via a multiple coarse-grained time series by the scale factor *τ*. For a one-dimensional time series of cumulative discharge rate {*x_i_*… *x_n_*}, MSE first constructs multiple coarse-grained time series by the scale factor *τ*. $y_{j}^{\left(\tau \right)}\;=\;1/\tau \sum_{i\;=\;\left(j-1\right)\tau +1}^{j\tau }x_{i},1\leq j\leq N/\tau$. * N* is the number of data points in the time series. For scale one, the time series {*y*^(1)^} is simply the original time series. The length of each coarse-grained time series is equal to the length of the original time series divided by the scale factor *τ*. Then, sample entropy was applied to the resulting coarse-grained time series plotted as a function of the scale factor τ, or MSE curve. The complexity of cumulative discharge rate was denoted as the areas (MSE_1–10_ and MSE_11–20_) under the MSE curve for short (1–10) and long (11–20) time scales. Each time scale was 10 ms, in correspondence with the down-sampling rate. The mathematical formula of sample entropy was $SampEn\left(m,r,N\right)=-\log\left(\frac{\sum_{i=1}^{N-m}A_{i}}{\sum_{i=1}^{N-m}B_{i}}\right)$, where *r* = 15% of the standard deviation of the force channel, *m* is the length of the template (*m* = 2), and *A*_i_ is the number of matches of the *i*th template of length *m + 1* data points, and *B*_i_ is the number of matches of the *i*th template of length *m* data points. *N* represented the number of data point of the time series (Pethick et al., 2015). A lower value represents greater regularity of force characteristics. In addition, we calculated a single coherence spectrum between a given pair of cumulative spike trains of five motor unit spike trains (Rosenbaum et al., 2011; Farina et al., 2016). The computations of pooled motor unit coherence were repeated and averaged across 500 random combinations of the single coherence spectrum from all identifiable motor units. The magnitude squared coherence values (C) were estimated with two unfiltered composite spike trains using a 1-s Hanning window and an overlap of 90% (Castronovo et al., 2015). The coherence values were converted to Fisher’s Z (FZ) values (Amjad et al., 1997) to minimize an intrinsic bias of the coherence estimates from each segment profile following corrective subtraction of the mean coherence between 100 Hz and 500 Hz (Castronovo et al., 2015). FZ value is formulated as: $FZ=\tan^{-1}\left(\sqrt{C}\right)$. Following the z transformation, we subtracted the mean coherence between 100 Hz and 500 Hz from each coherence profile because the mean coherence contained no significant coherence (Witte et al., 2007; Nawab et al., 2010). A pooled motor unit coherence, which reflects common drive to the motoneuronal pool in the spectral domain, contained two prominent spectral peaks in the range of 0–4 Hz (ZC_0–4 Hz_) and 13–35 Hz (ZC_13–35 Hz_; Hwang et al., 2017). All of the force variables and discharge variables of the three experimental trials in the control and EA conditions were calculated and averaged subject-by-subject. Signal processing and statistical analyses were completed using Matlab (Mathworks Inc., Natick, MA, USA).

### Statistical Analysis

The MVC values of index abduction of the young and older groups were compared with independent *t* statistics. With appropriate variable transformation to satisfy two-way repeated-measures analysis of variance (ANOVA) was used to examine the task effect (control vs. EA, within-subject factor) and group effect (young vs. older, between-subjects factor) on the task/force fluctuation variables, variables of stabilogram diffusion analysis, and motor unit discharge variables. The level of significance was 0.05. *Post hoc* testing was conducted in the presence of significant interaction or main effects. A particular interest of this study was differential modulations on force and motor unit behaviors between the young and older adults due to EA. Bonferroni corrections were used for determining the alpha level of significance for *post hoc* tests (*p* = 0.0125). Data are presented as group means ± 1 standard error of the mean. All statistical analyses were performed in IBM SPSS Statistics (v19).

## Results

### Task Error and Force Characteristics

Table 1 shows a summary of the results of performance variables for the young and older adults. Constant error was independent of both task and group effects (*p* > 0.05). In contrast, total error of force-tracking was subject to task and group effects (*p* < 0.05), with significant task × group interaction (*p* = 0.013). *Post hoc* analysis indicated that only the older group exhibited a greater total error in the EA condition than in the control condition (*p* = 0.002). Also, total error of the older group was larger than that of the young group in the EA (*p* < 0.001) condition. The size of force fluctuations was also subject to both task and group effects (*p* < 0.05), with a significant task × group interaction (*p* = 0.019). *Post hoc* analysis indicated that only the older group exhibited a greater size of force fluctuations in the EA condition than in the control condition (*p* < 0.001). The size of force fluctuations of the older group was larger than that of the young group in the EA (*p* = 0.001) condition.

**Table 1:** The results of analysis of variance (ANOVA) statistics to contrast performance variables between the young and old groups in the control and EA conditions.

Task performance		Control	EA	Statistics
Constant error	Young	−0.203 ± 0.041	−0.187 ± 0.027	Group: *F*_(1,26)_ = 0.41, *p* = 0.529, *η*^2^ = 0.015; Task: *F*_(1,26)_ = 0.01, *p* = 0.960, *η*^2^ = 0.003
(% MVC)	Old	−0.219 ± 0.063	−0.249 ± 0.042	Group × Task: *F*_(1,26)_ = 1.15, *p* = 0.293, *η*^2^ = 0.005
Total error	Young	0.492 ± 0.036	0.480 ± 0.032	Group: *F*_(1,26)_ = 9.72, *p* = 0.004, *η*^2^ = 0.261; Task: *F*_(1,26)_ = 5.29, *p* = 0.030, *η*^2^ = 0.017
(% MVC)	Old	0.687 ± 0.069	0.842 ± 0.101**	Group × Task: *F*_(1,26)_ = 7.18, *p* = 0.013, *η*^2^ = 0.023
RMS of force fluctuations	Young	0.404 ± 0.033	0.415 ± 0.033	Group: *F*_(1,26)_ = 7.94, *p* = 0.009, *η*^2^ = 0.221; Task: *F*_(1,26)_ = 7.88, *p* = 0.009, *η*^2^ = 0.032;
(% MVC)	Old	0.572 ± 0.064	0.753 ± 0.102***	Group × Task: *F*_(1,26)_ = 6.24, *p* = 0.019, *η*^2^ = 0.025;

Total error is jointly determined by constant error and the size of force fluctuations. **EA > control, p < 0.005; ***EA > control, p < 0.001.

Pooled linear-linear and log-log plots of stabilogram diffusion analysis are shown in Figures 2A,B, respectively. Table 2 summarizes the results of ANOVA statistics for all variables of stabilogram diffusion analysis. ∆*t_c_* varied significantly with manipulation of error factor (*p* = 0.001), and ∆*t_c_* of the older (*p* = 0.008) groups was smaller in the EA condition than in the control condition. $<\Delta F_{c}^{\text{2}}>$ and D_s_ were subject to a significant effect of task × group interaction (*p* < 0.05). *Post hoc* analysis further revealed that $<\Delta F_{c}^{\text{2}}>$ and D_s_ were differently modulated for the two groups. $<\Delta F_{c}^{\text{2}}>$ and D_s_ were greater in the EA condition than in the control condition only for the older adults ($<\Delta F_{c}^{\text{2}}>$: *p* = 0.004; D_s_: *p* = 0.001). H_s_ was a function of task effect (*p* = 0.005), and* post hoc* test indicated EA enhanced H_s_ of the older group (*p* = 0.002). None of the $<\Delta F_{c}^{\text{2}}>$, D_s_, and H_s_ of the young adults were dependent on the manipulation of EA (*p* > 0.05). Besides, D_l_ and H_l_ did not vary with the main effects and interaction effects of task and group (*p* > 0.05).

**Table 2:** Parameters of stabilogram diffusion analysis of static force tracking for young and old groups in the control and EA conditions.

		Control	EA	Statistics
∆*t_c_* (s)	Young	0.393 ± 0.016	0.359 ± 0.016	Group: *F*_(1,26)_ = 0.68, *p* = 0.417, *η*^2^ = 0.024; Task: *F*_(1,26)_ = 13.73, *p* = 0.001, *η*^2^ = 0.064
	Old	0.411 ± 0.024	0.369 ± 0.028^†^	Group × Task: *F*_(1,26)_ = 0.14, *p* = 0.711, *η*^2^ = 0.000
$<\Delta F_{c}^{\text{2}}>$ (%MVC^2^)	Young	0.401 ± 0.064	0.359 ± 0.016	Group: *F*_(1,26)_ = 1.18, *p* = 0.287, *η*^2^ = 0.038 ; Task: *F*_(1,26)_ = 4.37, *p* = 0.046, *η*^2^ = 0.051
	Old	0.397 ± 0.069	0.620 ± 0.084**	Group × Task: *F*_(1,26)_ = 5.71, *p* = 0.024, *η*^2^ = 0.067
D_s_ (%MVC^2^/s)	Young	0.587 ± 0.106	0.572 ± 0.106	Group: *F*_(1,26)_ = 1.36, *p* = 0.254, *η*^2^ = 0.043 ; Task: *F*_(1,26)_ = 6.87, *p* = 0.014, *η*^2^ = 0.079
	Old	0.582 ± 0.124	1.068 ± 0.191**	Group × Task: *F*_(1,26)_ = 5.77, *p* = 0.024, *η*^2^ = 0.067
D_l_ (%MVC^2^/s)	Young	−0.005 ± 0.004	−0.004 ± 0.003	Group: *F*_(1,26)_ = 0.01, *p* = 0.919, *η*^2^ = 0.000; Task: *F*_(1,26)_ = 0.78, *p* = 0.384, *η*^2^ = 0.028
	Old	0.000 ± 0.002	−0.008 ± 0.004	Group × Task: *F*_(1,26)_ = 2.55, *p* = 0.122, *η*^2^ = 0.092
H_s_ (%MVC^2^/s)	Young	0.939 ± 0.004	0.941 ± 0.004	Group: *F*_(1,26)_ = 0.51, *p* = 0.483, *η*^2^ = 0.018; Task: *F*_(1,26)_ = 9.17, *p* = 0.005, *η*^2^ = 0.050
	Old	0.938 ± 0.004	0.949 ± 0.005**	Group × Task: *F*_(1,26)_ = 0.51, *p* = 0.483, *η*^2^ = 0.020
H_l_ (%MVC^2^/s)	Young	−0.025 ± 0.009	−0.018 ± 0.009	Group: *F*_(1,26)_ = 0.35, *p* = 0.560, *η*^2^ = 0.012; Task: *F*_(1,26)_ = 0.44, *p* = 0.514, *η*^2^ = 0.014
	Old	0.001 ± 0.013	−0.024 ± 0.010	Group × Task: *F*_(1,26)_ = 3.55, *p* = 0.071, *η*^2^ = 0.112

∆t_c_, critical point of time; $<\Delta F_{c}^{\text{2}}>$, critical point of force fluctuations; D_s_, short-term effective diffusion coefficients; D_l_, long-term effective diffusion coefficients; H_s_, short-term scaling exponent; H_l_, long-term scaling exponent. ^†^EA < control, p < 0.01; **EA > control, p < 0.005.

### Discharge Patterns of Motor Units

The average numbers of decomposed motor units for older adults in the control and EA conditions were 26.7 ± 1.1 and 28.0 ± 1.4, respectively. The average numbers of decomposed motor units for young adults in the control and EA conditions were 27.0 ± 1.0 and 27.4 ± 1.2, respectively. Table 3 shows the statistical results of ANOVA for motor unit discharge variables. Both ISI_GAV_ and ISI CV_GAV_ were subject to marginally-significant effect of task × group interaction (ISI_GAV_:* p* = 0.058; ISI CV_GAV_: *p* = 0.050). To be prudent, *post hoc* analysis revealed that only the discharge variables of the older adults depended on the task effect, with significantly greater ISI_GAV_ (*p* = 0.005) and ISI CV_GAV_ (*p* = 0.007) in the EA condition than in the control condition. The discharge variables of the young adults were insensitive to the task effect (*p* > 0.05). ISI CV_GAV_ of the older adults was greater than ISI CV_GAV_ of the young adults in the EA condition (*p* = 0.005).

**Table 3:** Mean and standard errors of discharge variables for the young and old groups in the control and EA conditions.

Discharge property		Control	EA	Statistics
ISI_GAV_ (ms)	Young	56.69 ± 2.74	57.22 ± 2.86	Group: *F*_(1,26)_ = 1.74, *p* = 0.199, *η*^2^ = 0.060; Task: *F*_(1,26)_ = 5.60, *p* = 0.026, *η*^2^ = 0.024
	Old	59.55 ± 4.21	66.75 ± 4.39*	Group × Task: *F*_(1,26)_ = 3.93, *p* = 0.058, *η*^2^ = 0.017
ISI CV_GAV_	Young	0.215 ± 0.005	0.217 ± 0.005	Group: *F*_(1,26)_ = 4.40, *p* = 0.046, *η*^2^ = 0.113; Task: *F*_(1,26)_ = 4.46, *p* = 0.045, *η*^2^ = 0.104
	Old	0.219 ± 0.008	0.324 ± 0.025*	Group × Task: *F*_(1,26)_ = 4.21, *p* = 0.050, *η*^2^ = 0.099

ISI, averaged inter-spike interval for a single motor unit; ISI CV, coefficient of variance of inter-spike interval for a single motor unit. *EA > control, *p* < 0.01.

**Figure 4:**
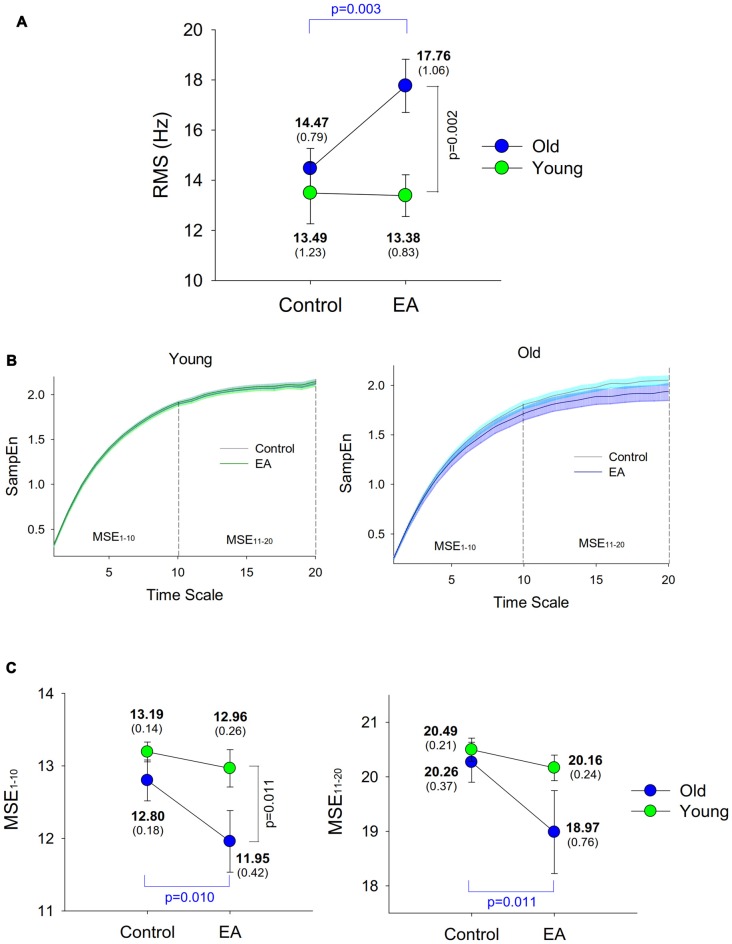
Population means and standard errors for parametric variables of cumulative discharge rate in the control and EA conditions. **(A)** RMS; **(B)** MSE; **(C)** MSE area of low (1–10) and high (11–20) time scales. Each time scale of the MSE curve is 10 ms, corresponding to down-sampling rate of 100 Hz.

Figure 3 shows a typical example of cumulative discharge rate from an EA trial of an older adult. The contrasts of size and complexity of cumulative discharge rate variability between the young and older adults in the control and EA conditions are shown in Figures 4A–C. In terms of RMS value, the RMS of cumulative discharge rate was subject to task (*F*_(1,26)_ = 4.95, *p* = 0.035, *η*^2^ = 0.163) and group (*F*_(1,26)_ = 5.93, *p* = 0.022, *η*^2^ = 0.058) effects, with a significant interaction effect of group and task (*F*_(1,26)_ = 5.61, *p* = 0.026, *η*^2^ = 0.066). *Post hoc* analysis revealed that RMS of cumulative discharge rate was greater in the EA condition than in the control condition for the older adults (*p* = 0.003), whereas the trend was not evident for the young adult (*p* = 0.920). The group effect was noted in the EA condition, and RMS of cumulative discharge rate of the older adults was larger than that of the young adults (*p* = 0.002). Figure 4B shows pooled MSE curves for young and older adults that represent the complexity of cumulative discharge rate variability of different time scales in the control and EA conditions. The short-time scale MSE (MSE_1–10_) was subject to group (*F*_(1,26)_ = 4.84, *p* = 0.037, *η*^2^ = 0.140) and task (*F*_(1,26)_ = 6.29, *p* = 0.019, *η*^2^ = 0.098; Figure 4C). Only the MSE_1–10_ of the older adults was suppressed with EA (*p* = 0.010) rather than MSE_1–10_ of the young adults (*p* = 0.459). In the EA condition, MSE_1–10_ of the older adults was smaller than that of the young adults (*p* = 0.011). The long-time scale MSE (MSE_11–20_) was subject to a significant task effect (*F*_(1,26)_ = 5.58, *p* = 0.023, *η*^2^ = 0.086) and a marginally significant interaction effect of task and group (*F*_(1,26)_ = 2.99, *p* = 0.056, *η*^2^ = 0.069), rather than group effect (*F*_(1,26)_ = 2.04, *p* = 0.165, *η*^2^ = 0.027). *Post hoc* analysis further revealed that MSE_11–20_ of the older group was lower in the EA condition than in the control condition (*p* = 0.011). MSE_11–20_ of the young group was not dependent on EA (*p* = 0.487).

**Figure 5:**
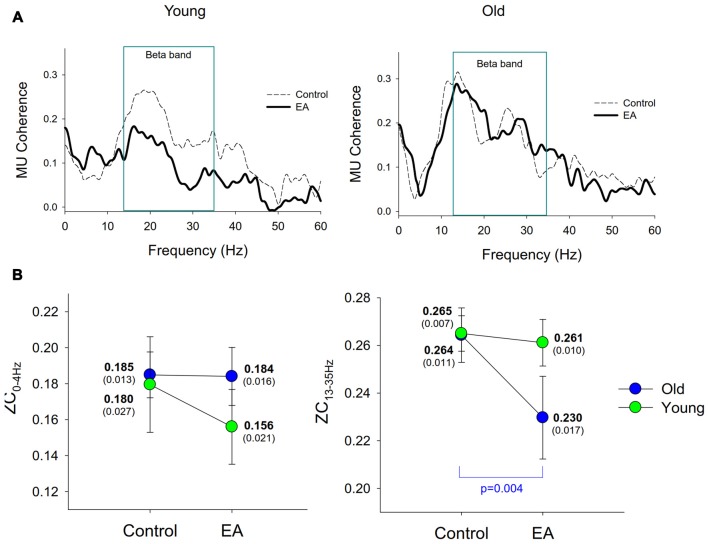
Discharge coherence among motor units. **(A)** The contrast of the pooled motor unit coherence between the control and EA conditions for the young and old groups. **(B)** The contrast of means and standard errors of the coherence peaks in the 0–4 Hz (ZC_0–4 Hz_) and 13–35 Hz (ZC_13–35 Hz_) bands between the control and EA conditions for the two groups.

Figure 5A contrasts the pooled motor unit coherence between the control and EA conditions for the young and older adults. The pooled motor unit coherence of the older adults was more modifiable to EA during force-tracking. The group effect of ZC_13–35 Hz_ was significant (*F*_(1,26)_ = 6.25, *p* = 0.019, *η*^2^ = 0.053), with marginally significant interaction of task × group interaction (*F*_(1,26)_ = 10.12, *p* = 0.056, *η*^2^ = 0.034) and insignificant group effect (*F*_(1,26)_ = 1.11, *p* = 0.310, *η*^2^ = 0.037). Only ZC_13–35 Hz_ of the older group was significantly suppressed by EA (*p* = 0.004), but not that of the young adults (*p* > 0.05; Figure 5B). However, ZC_0–4 Hz_ did not vary with the main and interaction effects of age and error size (*p* > 0.05).

## Discussion

The novel finding of this study was the age-related differential impacts of visual EA on static force-tracking with visual feedback containing higher-frequency error information. The high-frequency visual EA altered the strategic balance between the open-loop and closed-loop controls to guide static force-tracking. However, unlike the young adults, the older adults exhibited inferior task accuracy, greater motor unit discharge variability, lower complexity of discharge variability, and lower pooled motor unit coherence in the 13–35 Hz band in the EA condition than in the control condition. In contrast, the static force-tracking of the young adults was less affected by high-frequency visual EA in the behavioral and neurophysiological aspects.

### Age-Related Exacerbation of Task Precision with Error Amplification

It was clear that high-frequency EA had a negative impact on the older adults, who showed less task accuracy with the increased total error in the EA condition (Table 1). In contrast, the young adults were much less affected by EA. The age effect produced different impacts on task accuracy with EA, which were primarily attributable to the size of force fluctuations rather than to constant error (Table 1). Although the older adults became incapable of maintaining force steadiness with EA during force-tracking, the degree of force linear shifts from the target signal, which was greatly impaired for the elderly with the increased spatial resolution for representing the force on the monitor, was independent of the age effect and EA (Sosnoff and Newell, 2006; Kennedy and Christou, 2011). Hence, the age-related decline in force steadiness with EA in this study was not exactly attributable to excessive visuospatial information (Kennedy and Christou, 2011; Tracy et al., 2015), as the spatial resolutions of the monitor for the control and EA conditions were identical. Instead, we specified that the impairment of the EA-induced force steadiness of the older adults was a sequela of age-related increases in visuomotor loop delay, especially when the force-tracking control with EA relied comparatively on the feedback process (Chen et al., 2017). Perceived errors (or virtually-amplified errors) were more easily accumulated over time in the feedback system, resulting in fluctuating force outputs of the older participants (Miall et al., 1986, 1993). The evidence of a scheme switch during EA is further discussed in the following section.

### Visual Error Amplification and the Feedback-Prone Process

The relative significance of visuomotor control is modeled with a continuum regime from feedback to feedforward, depending on environmental contexts (Slifkin et al., 2000). A scheme switch in static force control was examined with stabilogram diffusion analysis, conceptually similar to the Hurst exponent. As force fluctuation dynamics could be modeled with a two linear regression lines in the stabilogram diffusion plot, the force fluctuation system should be regulated by two distinct processes (feedforward and feedback processes) in parallel with the postural control system (Collins and De Luca, 1993; Amoud et al., 2007). In this study, short-term scaling exponent (H_s_) was in the range of 0.5–1. Short-range force fluctuation system was persistent and subject to feedforward process, such that a high value in the force fluctuations was followed by another high value of force fluctuations. In contrast, long-term scaling exponent (H_l_) was smaller than 0.5. Long-range force fluctuations of the past and future were negatively correlated, predominated by a negative feedback loop.

The significant task effect of EA on critical point of time (∆*t_c_*) and critical point of force fluctuation $<\Delta F_{c}^{\text{2}}>$ (Table 2) implied an alteration in the steady-state force fluctuation behavior due to EA. It is known that ∆*t_c_* reflects the timing of stochastic activity when force fluctuations tend to drift away from an equilibrium point in an open-loop control mode before closed-loop feedback mechanisms dominate the force fluctuation behaviors. Therefore, a reduction in ∆*t_c_* in the EA condition indicated the effective range of the interval (0-∆*t_c_*) to regulate force fluctuations with the open-loop regime was shortened. Concurrently, the effective range of the interval (>∆*t_c_*), during which stochastic activity of force fluctuations exhibited a negative correlation between its increment (anti-persistence) due to feedback process, increased in the EA condition. The higher dependency on the feedback mechanism to stabilize static force in the EA condition is nicely compatible with our previous work for rhythmic force-tracking (Chen et al., 2017). Of note, unlike the young adults who showed a decreasing trend of $<\Delta F_{c}^{\text{2}}>$ in the EA condition, the elderly exhibited an EA-related potentiation of the critical point of force fluctuation ($<\Delta F_{c}^{\text{2}}>$; Table 2). The scenario suggests that closed-loop feedback of the older adults in the EA condition was called into play when the size of force fluctuation was greater than that in the control condition. The delayed feedback process might link to degenerated sensory systems to sensibly detect the force errors for the older adults. In effect, the strategy of taxing sensory feedback with EA was not suitable for the elderly because their degenerated sensory systems could also exaggerate feedback ambiguity and sensory conflicts (Bates and Wolbers, 2014).

According to the principle of stochastic resonance (Moss et al., 2004), noise is not always negative for a controlled process. Adding appropriate amount of noises can be helpful to perceive sensory signals incapable of being detected (substhreshold sensory information; Moss et al., 2004; McDonnell and Abbott, 2009). However, this study was not a case of noise benefits. All participants in this study were able to clearly detect execution errors on the monitor, and adding random noises could simply produce uncertainty of visuomotor control. In fact, the impact of uncertainty due to noisy visual errors should be minimized via filtering process. For instance, if error information contained high-frequency tremulous movements (such as 8–12 Hz physiological tremor) that were augmented via the EA process, error information above 0.4 Hz can hardly be responded with a feedback mechanism due to visuomotor delay (50–300 ms for humans; Miall et al., 1986, 1993; Joiner and Shelhamer, 2009). Moreover, according to the inhibitory deficit hypothesis, the elderly might fail to ignore functionally-irrelevant visual stimuli such as high-frequency errors (Van Gerven and Guerreiro, 2016). Namely, the noisy error feedback interfered with adjustments to tracking deviations. Moreover, the observation that EA increased the short-term effective diffusion coefficients (D_s_) and scaling exponent (H_s_) was noted only in the elderly (Table 2). Although it was not clear why the older adults exhibited a greater positive correlation between the past and future force fluctuation data in a short time interval, researchers who have used stabilogram diffusion analysis to investigate postural regulation attribute the parametric changes to stiffness increases in the ankle joint with the antagonist co-activity in compensation for muscle weakness (Collins and De Luca, 1995; Toosizadeh et al., 2015).

### Age-Related Variations in Discharge Behaviors Due to Error Amplification

In general, only the discharge behaviors and cumulative discharge rate of the older adults were tuned to EA; those of the young adults were not. For the elderly, high-frequency EA added to the motor unit discharge variability and inter-spike interval (Table 3). The motor unit discharge of older adults has been reported to be more susceptible to the amount of visuospatial information during a visuomotor task (Vaillancourt et al., 2002; Jordan et al., 2013). Consistent with this interpretation, high-frequency EA for the elderly presumably brought about more variant synaptic input (neural noises) to the motor neurons for visual-motor corrections. This argument was also supported by an age-related increase in RMS of cumulative discharge rate of the elderly in the EA condition (Figure 4A). On the other hand, the complexity of discharge variability was differently organized with respect to age and task. The lower short-time scale MSE_1–10_ for older adults (Figures 4B,C) collaborates the loss of complexity hypothesis with aging, although none of the previous studies have revealed this fact from complexity measures of motor unit discharge variability (Rey-Robert et al., 2011; Morrison and Newell, 2015). For older adults, it was evident that high-frequency EA gave rise to anomalies in the temporal organization of motor unit discharge dynamics, in view of a lower short-time MSE_1–10_ and long-time MSE_11–20_ in the EA condition than in the control condition. This scenario suggests declining capacity with aging to use the faster time scales of visual information (Sosnoff and Newell, 2008). When error size in visual feedback was amplified, the elderly failed to preserve stability and flexibility of motor unit discharge in face of the task constraint, because exaggerated error information challenged internal robustness of brain components that is less capable of compensating for increased processing noise with larger feedback delay times for the older adults (Heenan et al., 2014). Of note, the task-reverent complexity reduction in discharge variability of motor units for the young group was far from being evident. This argument is supported by the EA-related reduction in common oscillatory inputs in the 13–35 Hz band for the older group (Figures 5A,B). The β-band motor unit coherence reflects the interaction between cortical and spinal circuits, and its magnitude varies with the demands for sensorimotor integration. The β-band motor unit coherence has been showed to increase proportionally to greater amounts of visuospatial (Laine et al., 2014) and error information (Hwang et al., 2017) for young adults during a force-tracking task. Coincidentally, enhanced β-band electroencephalography (EEG)-EMG coherence is linked to steady-state force control with a higher degree of attentional focus (Kristeva et al., 2007; Witte et al., 2007). If the β-band motor unit coherence is a corollary to β-band EMG-EEG, the reduction in β-band motor unit coherence with EA implies that the older adults were distracted from performing constructive force-tuning. Besides, we could not exclude the possibility that an age-dependent variation in motor unit discharge was a physiological consequence of increasing the antagonist co-activity, in that older adults produce more motor overflow to the antagonists during a cognitively-demanding task than do young adults (Addamo et al., 2010). It was rational to observe longer ISI, larger discharge variability, and increasing D_s_ and scaling exponents with EA for the older adults.

### Methodological Considerations

First, EA is supposed to be an effective way to optimize static force-tracking for young adults (Hwang et al., 2017). However, the expected functional merits were not evident for the young adults because we introduced high-frequency force components (cut-off frequency: 20 Hz vs. 6 Hz, in this study vs. the previous study, respectively) and a larger EA factor (2 vs. 1.5, in this study vs. the previous study, respectively) to determine the size of the error feedback. Hence, the functional merits of EA for the young adults were partially counterbalanced by the increase in noisy feedback and excessive EA factor. Newt, one of the methodological advantages of using surface EMG decomposition is that it can detect more active motor units than intramuscular EMG can. Surface EMG decomposition has been adopted by numerous studies (De Luca et al., 2006; Hu et al., 2014; Martinez-Valdes et al., 2016; Chen et al., 2017; Hwang et al., 2017), though the decomposition accuracy among the various algorithms remains debatable (Farina and Enoka, 2011; De Luca et al., 2015b). It is beyond the scope of this study to resolve the issues of decomposition accuracy.

In conclusion, visual EA containing high-frequency error information degrades the static force control of the elderly due to ineffective information processing with the closed-loop system. The use of visual EA adds to neural noises, in light of the increase in the discharge variability of motor units. In reference to that of the control condition, the cumulative discharge rate of the older adults under the EA is more variant and less complex, in parallel with behavioral changes in tracking force. Collectively, variations in motor unit discharge due to augmentation of the virtual size of high-frequency error information links to a reduction in the common drive to the motoneuronal pool for force stabilization.

## Author Contributions

Y-CC and I-SH: conception or design of the work. LLL, C-LH: acquisition. I-SH, Y-TL, Y-CC: analysis. Y-CC, I-SH, LLL: interpretation of data. I-SH and Y-CC: drafting the work or revising it critically for important intellectual content. I-SH: final approval of the version to be published. Y-CC and I-SH: agreement to be accountable for all aspects of the work in ensuring that questions related to the accuracy or integrity of any part of the work are appropriately investigated and resolved.

## Conflict of Interest Statement

The authors declare that the research was conducted in the absence of any commercial or financial relationships that could be construed as a potential conflict of interest.

## Abbreviations

∆*t_c_*: critical point of time
<∆*F*^2^>: mean-squared value of the force fluctuations
$<\Delta F_{c}^{\text{2}}>$: critical point of force fluctuations
D_s_: short-term effective diffusion coefficient
D_l_: long-term effective diffusion coefficient
EEG: electroencephalography
EMG: electromyography
FZ: Fisher’s Z value
H_s_: short-term scaling exponent
H_l_: long-term scaling exponent
ISI_GAV_: grand average of mean inter-spike interval
ISI CV_GAV_: grand average for coefficient of variance of inter-spike interval
MSE: multi-scale entropy
MVC: maximal voluntary contraction
RE: real error
RF: real force
RMS: root mean square
T: target signal
VE: visualized error
VF: visualized force
ZC_0–4_ HZ: motor unit coherence peak in the range of 0–4 Hz
ZC_13–35_HZ: motor unit coherence peak in the range of 13–35 Hz
